# Developing a High-Quality Linkage Map for the Atlantic Killifish *Fundulus heteroclitus*

**DOI:** 10.1534/g3.119.400262

**Published:** 2019-07-09

**Authors:** Jeffrey T. Miller, Noah M. Reid, Diane E. Nacci, Andrew Whitehead

**Affiliations:** * Department of Environmental Toxicology, Center for Population Biology, Coastal and Marine Sciences Institute, University of California, Davis, CA; † Department of Molecular & Cell Biology, University of Connecticut, Storrs, CT, and; ‡ US Environmental Protection Agency, Office of Research and Development, National Health and Environmental Effects Research Laboratory, Atlantic Ecology Division, Narragansett, RI

**Keywords:** Atlantic Killifish, meiotic linkage map, RAD-Seq, genome synteny, scaffold inversions

## Abstract

Killifish (*Fundulus heteroclitus*) are widely distributed among different aquatic environments where they demonstrate an impressive range of highly-plastic and locally adaptive phenotypes. High-throughput sequencing has begun to unravel the mechanisms and evolutionary history of these interesting features by establishing relationships in the genotype-phenotype map. However, some genotype-phenotype analyses require a higher order of contiguity than what initial scaffolded (fragmented genome assembly where contigs have been assemble into scaffolds) genome assemblies can provide. Here, we used 5,685 high-quality RAD-Seq markers from a single mapping family to order 84% of the scaffolded genome assembly to 24 chromosomes. This serves to: 1) expand the killifish genomic toolkit, 2) estimate genome-wide recombination rates, and 3) compare genome synteny to humans and other fishes. After initially building our map, we found that the selection of thresholds for sequence data filtration highly impacted scaffold placement in the map. We outline each step of the approach that dramatically improved our map to help guide others toward more effective linkage mapping for genome assembly. Our final map supports strong conservation of genomic synteny among closely related fish species and reveals previously described chromosomal rearrangements between more distantly related clades. However, we also commonly found minor scaffold misorientations in *F. heteroclitus* and in other assemblies, suggesting that further mapping (such as optical mapping) is necessary for finer scale resolution of genome structure. Lastly, we discuss the problems that would be expected from misoriented/unplaced scaffolds and stress the importance of a quality mapped genome as a key feature for further investigating population and comparative genomic questions with *F. heteroclitus* and other taxa.

Understanding evolution, and how it generates taxonomic and phenotypic diversity, requires tools that can examine now genetic variation is shaped by the environment. Killifish (*Fundulus* sp.) form a model study system that is well-suited for such studies because they occupy a wide range of naturally variable and human-altered environments. A significant and growing body of research has documented locally-adaptive and plastic phenotypes that have been shaped by temperature, hypoxia, salinity, and pollutants (Burnett *et al.* 2007). Population genetic and gene expression analyses with killifish have revealed some of the genomic features and mechanisms that underlie these phenotypes (*e.g.*, Brennan *et al.* 2015; Healy *et al.* 2010; Nacci *et al.* 2016; Oleksiak *et al.* 2005, 2011; Whitehead *et al.* 2011). This work has been facilitated by the development of genomic tools for killifish, which now includes an annotated Atlantic killifish (*Fundulus heteroclitus*) reference genome assembly (Reid *et al.* 2016, 2017). The *F. heteroclitus* genome has been available as an un-mapped assembly that is sufficient for some analyses, such as a read mapping template for RNA-seq and population genomics (genomic DNA re-sequencing). However, some types of analyses, such as genotype-phenotype mapping or comparative analysis of genome structure, require a greater level of chromosomal contiguity than that which most draft genome assemblies can provide (Lewin *et al.* 2009). Mapping the killifish genome assembly into chromosomes enables additional research questions to be addressed and furthers the genetic toolkit available for this model species.

Initial *de novo* genome assemblies are typically composed of many fragments of varying sizes (scaffolds) due to the difficulty of assembling across highly repetitive regions of the genome, which is particularly challenging for assemblies that are derived from short-read sequences (Treangen and Salzberg 2012). To increase contiguity of a reference genome, scaffolds can be mapped to a position and orientation in a chromosomal-level assembly with physical mapping (such as Hi-C and Optical Mapping) and genetic mapping (Linkage or Recombination Mapping). Physical mapping resolves a large proportion of reference genome scaffolds to their physical position in one or few individuals (Schwartz *et al.* 1993; van Berkum *et al.* 2010). In contrast, recombination mapping relies on the statistical detection of linkage between genetic markers among related individuals (Sturtevant 1913). Inferring a relative position and orientation for scaffolds with recombination mapping inherently includes recombination rate estimates along the genome. Recombination rates are particularly useful for modeling demographic and evolutionary processes that affect the fate of population genetic variation. We chose linkage mapping to 1) increase the contiguity of the *F. heteroclitus* reference genome and to 2) obtain local recombination rates for the species.

Initially, we applied common filter parameters to our sequence data, which resulted in a low-quality map that commonly split up scaffolds and resulted in unreasonably high recombination rates. To determine if the use of arbitrary parameters had influenced the map quality, we applied a series of custom filters that were optimized for our data. We optimized the threshold parameters of our filters by visualizing the empirical distribution of the sequence data at each filter step. Our data-guided filters and agglomeratively clustering linked scaffolds (merging smaller clusters) built a male and female map that were more congruent with one another than in the initial map, and more congruent with the other mapped fish genomes. We merged these maps to correct and extend the previous *F. heteroclitus map* and to evaluate the quality of the final map. We predicted that our final map was of high quality if it tended to have conserved gene synteny with closely-related fish genomes (Kai *et al.* 2011), and if we recovered the well-characterized chromosomal rearrangements that have been observed in more distantly related taxa (Braasch *et al.* 2016). We found that the quality of our map improved with our heuristic approach to minimizing the retention of deeply covered markers that contained genotyping errors and by minimizing the influence of spurious linkage caused by missing data.

Much of our approach to improving our map consisted of routine, data-guided techniques that have been applied by others or implemented in software packages. However, it is often unclear how and why important threshold parameters are set. We have outlined our heuristic approach for marker selection to provide a simple guide for using basic visualizations of the sequence data to discover the dataset-specific filter parameters. These parameters are discussed in the results and discussion section. Lastly, we use the killifish map to highlight some interesting variation in synteny, and briefly discuss the limitations of mapped assemblies when inferring fine-scale structural evolution of chromosomes.

## Material and Methods

### Samples, sequencing, alignment and variant calling

A single mapping family was generated from a mating between an outbred male and female *F. heteroclitus*. The male and female founders were sexed by the presence of secondary sex characteristics; males exibit a colorful speckled patterns while females are drab and become visibly gravid when reproductively active (Abraham 1985). Parents were collected from Bar Harbor, ME, in 2007, held under clean laboratory conditions and mated in 2012, producing ∼150 larval siblings. After hatching, genomic DNA was isolated from 90 of these and both parents with the Qiagen DNeasy kit and digested with SbfI restriction enzyme. 96 unique custom barcodes were ligated to genomic DNA of the 90 offspring and parents, with parents represented on 3 different barcodes each (File S1). Barcodes were distinguished by at least two mutations to prevent erroneous read assignments. A total of two lanes of 100 nt paired-end Illumina HiSeq 2500 sequences (268,373,087 + 360,771,323 reads) passed quality filters. Reads were de-multiplexed and counted per barcode in an initial lane of sequencing to determine relative concentrations of each individual with the pool. Individual samples were re-pooled with readjusted concetrations in the second lane of sequencing to normalize the number read per barcode. Reads with low quality, without a RAD site, or an un-matched barcode were discarded. Barcode and low-quality sequences (below phred score of 10 per 15 bases) were trimmed from the sequences and sorted with custom perl scripts (File S2). Eleven and 23 million reads were assigned to the parents (female and male respectively), leaving just fewer than 600 million reads distributed among 90 sibling offspring (average of 66.6 thousand reads per offspring). These reads were aligned to the *F. heteroclitus* reference genome (version 3.0.2, NCBI RefSeq assembly accession: GCF_000826765.1) using BWA (Li and Durbin 2009). Duplicate reads were marked with SAMBLASTER, and all reads were converted to sorted BAMs with SAMTOOLS (Li and Durbin 2009; Faust and Hall 2014). We used SAMTOOLs to calculate the depth of coverage per base for visualization and pre-genotype caller filtration as discussed in the results (custom R scripts, File S3). Alignments (BAM files) that met the pre-filter criteria were passed to FreeBayes, a haplotype-based Bayesian genotype caller, using the four best alleles per locus with the ‘pooled discrete’ flag (Garrison and Marth 2012). BAMTOOLS marked low mapping quality reads (x < 30, reflects base call quality, depth, and possible number of alignments) and improperly paired reads for removal (Barnett *et al.* 2011).

### Filtering variants for quality genotypes

We read the VCF file from Freebayes into R to extract coverage statistics for bi-allelic single nucleotide polymorphisms (SNPs). As discussed further below, we visualized sequence coverage and set filter thresholds to exclude sites with unusually high coverage, and sites/genotypes with coverage too low to produce an accurate genotype call (Table 1, Figure 2). After an intial filter that removed sites with extremely high coverage, we then filtered sites where the depth of the major and minor alleles were highly imbalanced. We visualized allele balance by plotting the ratio of coverage and setting filters to exclude sites where the ratio highly deviated from a bi-allelic segregating site (1:3, 1:1, or 3:1). Next, we set individual genotypes with very high and low coverage to “missing”, and filtered sites with less than 50 of the 90 possible genotypes. With these markers, we imputed the most likely parental genotypes from the offspring genotype segregation ratios. This step allowed us to filter and correct offspring genotypes according to the observed parent genotypes. For bi-allelic SNPs, there are 4 possible informative genotypic combinations within a cross of two outbred parents (aa x ab, ab x ab, aa x bb, or ab x bb). To call the most likely parental and offspring cross-type for each locus, we used Maximum Likelihood (ML). We assumed the genotypes followed a multinomial distribution with a global heterozygote dropout error rate *e*. Under this model, the likelihood of an error rate *e* and parental cross *aa x ab* given a distribution of genotypes *n_aa_,n_ab_,n_bb_* would be $L\left(e,aaxab|n_{aa},n_{ab},n_{bb}\right)={\left(0.5+\frac{1}{2}e\right)}^{n_{aa}}+{\left(0.5-e\right)}^{n_{ab}}+{\left(\frac{1}{2}e\right)}^{n_{bb}}$. The same logic follows for the other cross-types. We estimated the global ML value of *e* and the cross-type for all loci simultaneously using the custom R functions in File S3. We determined that roughly 3,000 impossible offspring genotypes should be corrected to heterozygotes. These genotypes were due to allelic dropout, where sequencing had not sampled both alleles of a truly heterozygous individual. An example of this is if parental genotypes are het/homo (aa x ab) cross-type, but one or more offspring were identified as homozygous alternate (bb) at that locus; genotypes of these individuals would have been corrected to ab.

**Table 1:** Criteria for filtering loci and genotypes. Filter steps that included a vizualiztion for setting thresholds are labled with a letter that corresponds to Figure 2. Unlabled filter steps did not require a visualiztion

	Filter	Loci	Genotypes	% missing	Criteria
**A**	Coverage Per Base in Ref. Genome	**89,573**	**7,264,996**	**9.88**	Pre-genotyping filter (very low and high cov.)
**B**	Locus Read Count Ratio	**71,762**	**5,817,163**	**9.93**	Allelic bias > than 6/1 removed
**C**	Locus Total Coverage	**39,030**	**3,465,323**	**1.35**	Removed markers > 1400 and < 320 reads
**D**	Genotype Coverage	**39,030**	**1,972,374**	**43.85**	Genotypes < 8 and > than 40 reads to missing
**E**	Genotype Read Count Ratio	**39,030**	**1,944,174**	**44.65**	Allele bias in heterozygotes > (−/+ 3.26 log2)
**F**	Locus Missing Genotypes	**24,227**	**1,478,991**	**32.17**	Removed loci with fewer than 50/90 genotypes
	Correct Allelic Dropout	**24,227**	**1,478,991**	**32.17**	Impossible genotypes corrected
	Segregation Distortion	**20,093**	**1,226,282**	**32.19**	Binom/chi square to remove segregation distortion
	Parent-Offspring Match	**19,928**	**1,216,601**	**32.17**	ML cross-type of offspring ≠ parents

After these corrections, we evaluated each locus for segregation distortion from the most likely cross (aa x ab, ab x bb, and ab x ab) with binomial and chi-square tests (for single and double heterozygote cross-types, respectively) and excluded each locus with resulting p-values < 0.05. For double heterozygote cross-type markers (ab x ab), all three genotypes are possible in offspring, which prevents the correction of impossible genotypes and reduces the power for detecting segregation distortion. We excluded these loci from the marker set for this and other reasons discussed below. Finally, sites were excluded when the parent genotypes and ML offspring cross-type did not match. This final set of markers was used for the linkage mapping.

### Assignment to linkage groups and mapping

To establish the initial linkage groups, we calculated the average pairwise recombination frequencies for all scaffolds with the R ONEMAP package (Margarido *et al.* 2007). Each scaffold (maximum size ∼6mb) containing more than one locus of a given cross-type was checked for contiguous regions of unlinked loci to identify potential misassembled scaffolds. Initial agglomerative clustering grouped scaffolds with recombination frequency less than 0.07 with any scaffold in the group. Pairwise recombination frequencies grouped the initial LGs showing evidence of linkage and excluded a small number of individual loci showing strong evidence of linkage to multiple groups (agglomerative clustering).

To further imporve the computational efficieny of the marker ordering step, we downsampled markers and generated a map for each of the parents. The parent-specific maps (called sex-specific maps here) were generated by separating markers by the ML cross-type that we determined for each marker in the previous filtration steps. As indicated above, double heterozygote cross-type markers (ab x ab) cannot be filtered or mapped by the same criteria as the sex-specific cross-type markers. We discarded double heterozygote cross-type markers and retained the two sets of sex-specific cross-type markers (*aa x ab* and *ab x bb*). Sex-specific cross-type markers are informative for calculating recombination in each of the parents; recombination rate is calculated from the pairs of markers that are heterozygous in one parent and invariant in the other. We then downsampled these markers to the 5 most complete markers on each scaffold. Markers assigned to each linkage groups were loaded and mapped independently in JOINMAP (maximum likelihood-mapping algorithm and default parameters) (Stam 1993; Wu and Ma 2002). ALLMAPs merged the sex-specific maps to form the final consensus map (Tang *et al.* 2015). ALLMAPs uses a ‘traveling salesman problem’ solver to compute a consensus scaffold order (rather than marker order) and orientation from the equally weighted sex specific maps based on scaffold ID and SNP position (Figure S1). ALLMAPS then generates updated FASTA, agp, and CHAIN files with the un-mapped genome assembly and the consensus map. This CHAIN file was used with LIFTOVER (Hinrichs 2006) on the NCBI *F. heteroclitus* gene model set (NCBI gff version 3.0.2) to map genes to their new chromosomal map position for gene synteny analysis. To validate the final order of scaffolds, we evaluated the co-linearity of the sex-specific maps and final concensus map.

To align the consensus map LG labels with a previous *F. heteroclitus* linkage map, 240 microsatellites and SNP markers from (Waits *et al.* 2016) (called ‘microsat map’ hereafter) were blasted to the NCBI reference genome to match the microsat markers to the scaffolds that we mapped. We also blasted the microsat markers to the platyfish genome to find the ‘best-hit’ chromosome and determine if linkage group assignments in both maps were supported by chromosomal homology between platyfish and killifish. We renamed our linkage groups to reflect the microsat map LG names where possible and compared the resulting orders of both maps.

### Orthology and synteny analysis

**Figure 1:**
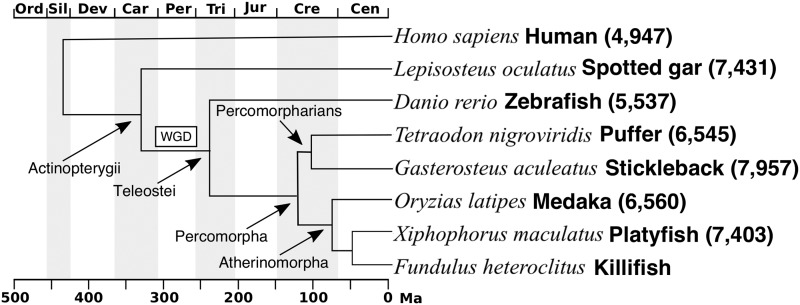
Phylogenetic relationships among species used in our comparative analyses. Atherinomorphs include Southern platyfish *X. maculatus*, and Japanese medaka *O. latipes*. Percomorpharians include green-spotted puffer *T. nigroviridis* and three-spined stickleback *G. aculeatus*. Teleostei also includes zebrafish *D. rerio*. A whole genome duplication (box marked WGD) occurred in the teleost lineage after it diverged from Actinopterygii that includes the spotted gar *L. oculatus*. Phylogeny is adapted from Betancur-R *et al.* (2013). The number of 1-to-1 orthologs included in the synteny analysis is denoted in brackets.

Gene positions and karyotypes from each species in the analysis were derived from publicly available genome feature format files (Figure 1, File S4). One-to-one orthologous genes were selected for this analysis from an orthology database (File S6) for a number of species generated by a reciprocal blast algorithm (Li *et al.* 2003; Reid *et al.* 2017). From this list of species, genomes were selected for synteny analysis if the publicly available genome feature format file was mapped to chromosomes. Whole genome ortholog oxford plots were visualized in R. We visualized the order of scaffolds in the male and female generated linkage maps and compared 1-to-1 ortholog synteny between *F. heteroclitus* and each species with CIRCOS (Krzywinski *et al.* 2009). We used these comparisons to designate homologous chromosomes between killifish and other species. For a complete set of figures showing homology, see File S6.

### Data availability

All data were deposited under NCBI BioProject PRJNA177717 (Fundulus_heteroclitus-3.0.2). Demultiplexed sequence data are submitted at NCBI Sequence Read Archive (SRA, submission: SUB5220906). The map that orders scaffolds in the *Fundulus heteroclitus* reference genome assembly is deposited at EMBL-EBI Biostudies (accession S-BSST163). Supplemental files and figures are available at FigShare. File S1 is a list of barcodes for sequence libraries. File S2 is the custom perl scripts for demultiplexing sequence libraries. File S4 are custom R scripts for data filtering. File S4 contains mapped genome data and orthology tables. File S6 contains circos images of chromosomal homology for all taxa in this analysis. Supplemental material available at Figshare: https://doi.org/10.25387/g3.8001572.

## Results & Discussion

A previous genetic map with 240 markers ordered ∼25% of the genome sequence into 24 linkage groups (LGs) (Waits *et al.* 2016). We sought to extend this microsatellite-based map and include more of the genome to improve the killifish genomic toolkit and compare genomic synteny to humans and other fish. We used Restriction Site Associated DNA Sequencing, or ‘RAD-Seq’ (Miller *et al.* 2007), markers from a single family including 90 larval F1 offspring and their parents (Baird *et al.* 2008; Baxter *et al.* 2011) to map the genome assembly of *Fundulus heteroclitus*. Our heuristic approach resulted in a recombination map of 5,685 RAD-Seq markers (mean density of 6.6 markers per Mb) that ordered 1,287 of the largest (216/221 of the scaffolds >N50) of the 10,180 scaffolds. The ordered scaffolds account for ∼84% of the 1.02GBs and gene models in the current reference assembly. In total, this map ordered 854 MB of genomic sequence into chromosomes and provide recombination rate estimates along each chromosome.

### Strategies for building a good map

**Figure 2:**
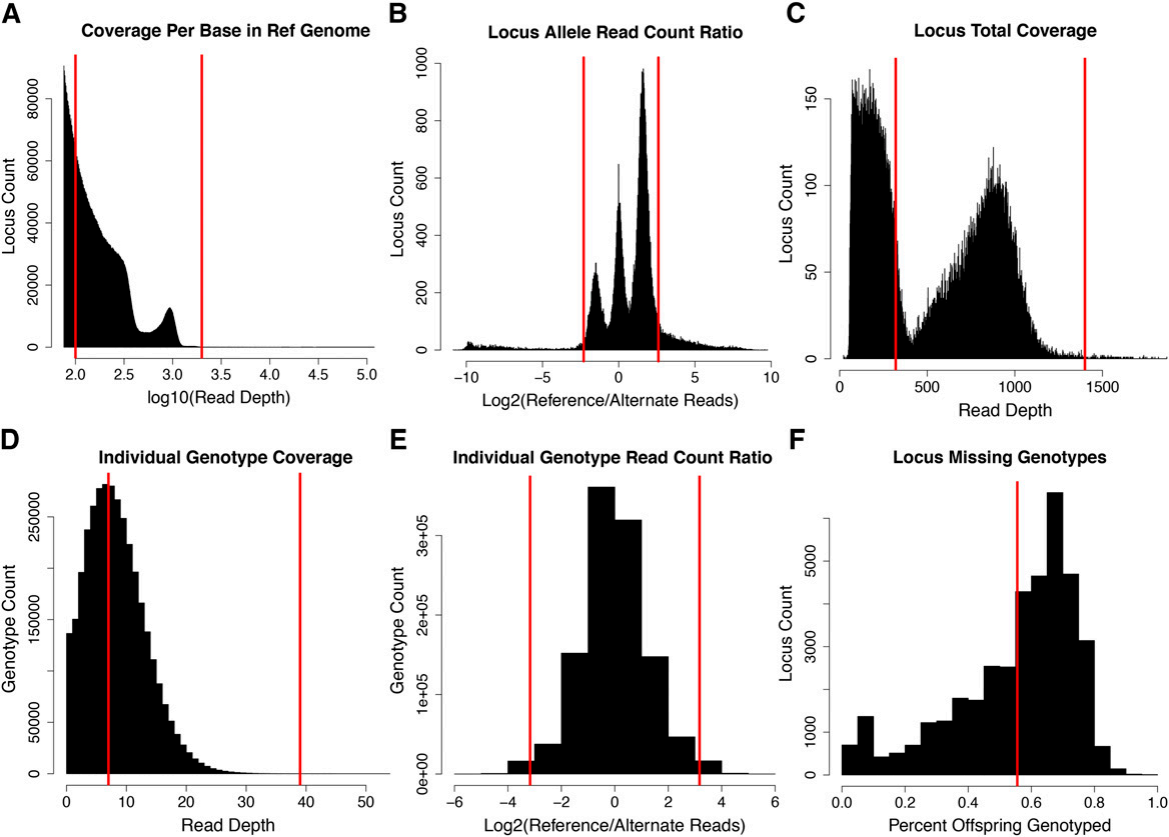
Designation of data filter thresholds guided by visualization of sequence/allele coverage and missing data distributions. Thresholds for our data were determined with simple visualizations of allele and sequence depth for loci and genotypes. Cutoff values at each filter step are indicated on plots with red vertical lines. Filters are for entire loci or individual genotypes (column 1). Each panel (A-F) corresponds to the filter in Table 1. These thresholds are specific to our data and are aimed at reducing genotype errors that are expected in reference genome alignment. Mapping algorithms are sensitive to erroneous genotypes and their removal increased the co-linearity of genomic scaffolds and markers in each of our maps that were estimated from recombination in either parent. We set these thresholds by visualizing: A) The log10 coverage for each base in the reference genome (depth > 75). B) The log2 ratio of read counts for the major and minor alleles for each locus. C) The distribution of total coverage for each locus summed across all individuals. D) The distribution of individual genotype coverage (major + minor allele). E) The log2 ratio of major to minor alleles for individual heterozygote genotypes. F) The distribution of missing data in remaining loci (percent of offspring genotyped).

The proportion of quality markers in a given sequence dataset is influenced by many factors, including the quality of the reference genome assembly, the sequencing platform, marker depth and density, and heterozygosity of the sequenced samples (Song *et al.* 2016). Therefore, the optimal threshold parameters for marker selection would be expected to vary between datasets. Initially, we designated our depth and segregation distortion filters for our map by surveying the literature for maps with similar taxa and experimental designs. Our sequence data were of relatively low coverage, so applying conservative and inclusive parameters that we surveyed resulted in either a low proportion of genome in the final map, or the appearance of incongruities with other maps. A heuristic, iterative approach guided by a series of data visualizations addressed these issues by optimizing thresholds to minimize the number of genotyping errors (due to artifacts such as repetitive sequence and paralog alignment) and missing data, while retaining the highest number of informative markers. As a result, the correlation with the previous *F. heteroclitus* map, between our male and female map, and between the *F. heteroclitus* map and closely related species had improved. We discuss the rationale for each of our steps to guide threshold parameter discovery for others (Table 1, Figure 2).

### Marker selection with basic visualizations of the data

Parameter selection can be cumbersome with a large number of densely spaced markers. We found that filtering the alignment before genotyping reduced the dataset to a more manageable size for faster re-analysis and data visualization. BAMTOOLS can quickly generate a two-column file of depth and position for easy loading and visualization in R. The initial per base alignment depth ranged from 1 to well over 100,000 reads (Figure 2A). Visualizing the depth allowed us to set an upper threshold for coverage just beyond the accumulation of deeply covered positions in the genome, which is consistent with intended targeting of sequencing at RAD tags (centered near log10 = 3.0, or 1000). We suspected that the very widely distributed and more deeply covered sequence was the result of highly-repetitive sequence alignments that are found throughout the genome and are not reliable markers for recombination mapping (Treangen and Salzberg 2012) (Figure 2A, red line set at log10(depth)=3.2). The distinct accumulation of low coverage positions is a combination of paired-end read alignments and probably some non-RAD tag associated reads (Figure 2A, below red line at log10(depth)=2). Our lower threshold was more inclusive than would be necessary to discover an informative mapping marker (100x across 92 individuals). While this filter step is redundant to a later filtering step aimed at suspicious genotypes and markers, the primary purpose here was to reduce the dataset size. We found that pre-filtering the very high and very low coverage sequence sufficiently reduced the time and computational load of later genotype calling and visualization. This step would be particularly useful for large datasets or genotype callers that require greater computational resources.

After calling genotypes, we filtered the data based on visualizations of allele coverage, balance, and depth distributions of loci and called genotypes. The first filter removed markers with a highly skewed ratio of reference to alternate allele reads (minor/major allele coverage < 0.167). Even though short read sequences have a low sequencing error rate, highly skewed ratios could indicate a small number of sequencing errors at an invariant site. These sites are more likely to be additional alignment errors where multiple loci have mapped to a single locus in the reference genome but had lower coverage than the pre-genotype filter threshold. The alignment error rate is expected to vary between genome assemblies and taxa; alignments to genomes with expanding gene families or genomes that have more recently experienced a whole genome duplication (such as teleosts compared to gar, Figure 1) would be expected to have an elevated rate of mis-aligned and ambiguously mapped reads. This threshold applied to our data (Figure 2B red line at ∼6:1 and 1:5, ref/alt) eliminated 18,000 of almost 90,000 loci (Table 1). The asymmetry between the peak heights of heterozygotes in this distribution (left and right peaks in Figure 2B) is due to the tendency for the alternate allele (relative to the reference genome) to be at lower frequency in the parents (ex: parents are more likely to be ref/ref X ref/alt than ref/alt X alt/alt). The increase in the number of loci in the positive tail (rightmost peak) is due to alignment bias toward reads that contain more reference alleles (Brandt *et al.* 2015). We chose a slightly offset threshold in the positive direction (log2(ref/alt) =2.6) to avoid removing a large number of quality markers that would be removed with the symmetrical threshold of log2(ref/alt) =2.3.

The total locus depth distribution (now for genotypes) guided the choice of thresholds to capture what we suspected as the range of coverage for RAD tags (Figure 2C). Loci with coverage less than 320 and higher than 1,400 reads were eliminated from the analysis, removing 32,000 and 116 loci respectively. While this may appear aggressive, loci with 320 reads split between half of the offspring would, at best, provide ∼7 reads for each genotype (3.5/allele). We later determined the lower threshold of 7 reads by comparing the proportion of parent genotypes that matched with the ML parent genotypes as determined from the offspring genotype frequencies (Figure 2D; red line at 7). Below 7 reads, the proportion of markers matching expectations fell sharply. We also used the ML genotypes to remove markers that differed from Mendelian expectations (segregation distortion). Genetic mapping tools commonly provide similar filters for markers with genotype frequencies that deviate from segregation expectations in a bi-parental cross. Real segregation distortion is common in mapping populations (Xu 2008). Our ML approach likely included some distortion and used an allelic-dropout error term (see Methods) in an effort to retain as many markers as possible. The R code for inferring the ML parental genotypes from offspring is relatively straightforward and is included in the supplemental File S4. The upper threshold at 40 reads per individual genotype excluded a very small number of highly covered genotypes (Figure 2D; red line at 40).

Our last set of visualization-guided filters removed individual genotypes that had suspiciously high or skewed coverage. We observed a small number of genotypes with suspiciously high allelic bias (10:1 major/minor allele) among the genotypes that remained after prior filtering. These genotypes (∼1%) were also changed to missing to reduce the possibility of retaining sequencing errors that changed the genotype call (Figure 2E). Removal of genotypes in these two steps increased the proportion of missing data to ∼45% (Table 1), indicating that many of the remaining loci had very low coverage (likely falling in the paired-end of reads). As discussed below, markers with a higher proportion of missing data tend to spuriously link to multiple linkage groups. High proportions of missing data can lead to errors in marker order and tend to deflate mapping distance (Hackett and Broadfoot 2003). We filtered out markers with more than 50/90 missing genotypes (>55.55% missing), which resulted in 24,227 higher coverage loci (Figure 2E). We used parental genotypes to determine that 2,998 individual offspring genotypes were incorrectly called homozygous alternate, presumably due to allelic dropout. Correcting genotypes affected by dropout rescued just over 10,000 loci from the following segregation distortion filter (*P* < 0.05, exact binomial and chi-square tests). We corrected dropout genotypes to heterozygotes and re-filtered the data for markers where the parent and the ML determined parent genotypes mismatched.This mappable set of 19,928 markers from the heuristic filtering step aligned to 1,287 assembly scaffolds that accounted for ∼85% of the killifish genome sequence (854 Mb of 1,021.88 Mb) and gene models (21,435 of the 25,601 NCBI gene models).

### Assignment to linkage groups

Markers are typically assigned to linkage groups by varying the recombination frequency and LOD threshold parameters *ad hoc*, and manually assigning groups of markers with one another. During the assignment step, we found that markers with a greater proportion of missing data were susceptible to spurious linkage with markers on different linkage groups. That is, clustering markers without considering scaffold ID information tended to link scaffolds that otherwise appear unlinked when considering all markers on a scaffold. We cannot identify these markers *a priori*, and once anchored in their correct LG, spuriously linked markers can still contain information on the order and orientation of scaffolds within LGs, so it may be beneficial to retain them. To keep these markers, we took an iterative approach that clustered entire scaffolds of markers together. We calculated the pairwise recombination fraction for markers to perform the agglomerative grouping and validated our strategy of assigning scaffolds to linkage groups by visualizing the matrix of recombination frequencies for markers along each scaffold (Figure S2).

Our initial filtering focused on removing low quality markers. Even though the remaining markers are of higher quality, many are redundant to one another and do not provide additional mapping information. After scaffolds were clustered into linkage groups, we added another filtering step to reduce these redundant markers to improve mapping computational efficiency. Before assigning markers it linkage groups, the final marker set was pruned to the 5 RAD loci per scaffold. Markers that are in close proximity to one another, such as those in a single RAD-Tag, are extremely unlikely to contain a recombination event. As such, these nearby markers do not add to the resolution of the final map but do significantly increase the computation time for mapping. Rather than drop non-recombinant or random markers, we thinned the dataset to five markers per parental cross-type per scaffold to reduce the mapping computation time without sacrificing resolution. As a final step to conservatively filter out suspicious genotypes, we removed a small number of loci that appeared as short double crossover events within a scaffold (visualized in Joinmap) in one or more individuals, bringing the total number of markers to 5,739 (2,992 and 2,855 male and female specific markers, respectively: Table 2).

**Table 2:** Summary statistics for the male, female, and consensus map of *Fundulus heteroclitus*

	Pre-Map	Female	Male	Merged Female and Male Map		
Linkage Groups	Scaffold N50 1.25 Mb	24	24	Anchored	Oriented	Unplaced
Markers (unique)		2,809	2,930	5,685	3,733	54
Markers per Mb		3.5	3.7	6.6	6.1	0.3
Scaffold L50	221	213	212	216	169	5
Scaffolds (total)	10,180	1,027	1,055	1,287	571	8,893
1 marker Scaffolds	NA	411	367	380	0	47
2 marker Scaffolds		145	178	173	60	2
3 markers Scaffolds		91	132	111	61	1
>=4 markers Scaffolds		380	378	623	450	0
Percent assembly		78.4	78.1	83.7	59.4	16.3
Total bases (Mb)	1021.88	800.77	798.35	854.98	607.44	166.92

### Ordering within linkage groups and the final Map

Once markers are assigned to linkage groups, the outcrossing mapping algorithm typically integrates the two sex specific maps into a single consensus map (Wu and Ma 2002). We chose a different approach. The outcrossing algorithm relies on the parental double heterozygote markers as landmarks for a map that integrates the sex specific maps. We found that this approach resulted in a final map where the scaffolds were more commonly broken within the group (mapped to different positions along the linkage group rather than clustered), which appeared to increase the uncertainty about the consensus position for each scaffold. These issues could be related to the reduced power to detect segregation distortion in double heterozygote markers, and to the inability to correct allelic dropout and impossible genotypes. To explore this issue further, we re-mapped two sets of markers for each linkage group; we separated markers that were informative for recombination in each parent to generate the sex specific maps independently (see methods for clarification). We found that the sex specific maps were congruent with one another and were far less likely to separate markers on each scaffold. We decided to merge these maps with ALLMAPs (Tang *et al.* 2015, Table 2). The ALLMAPs ordering algorithm improved the congruence between the individual maps and merged map by anchoring whole scaffolds from the marker data rather than treating each marker as an independent observation. A drawback to this approach is that scaffolds that have evidence for mis-assembly (chimeric scaffolds that were improperly joined during the primary genome assembly phase) are anchored to a common position. Chimeric scaffolds could be addressed before the mapping stage by evaluating recombination frequencies within scaffolds and splitting the groups of markers that have a discordant ratio between physical distance and recombination rate. ALLMAPs includes a resource for merging maps with scaffolds that were split because they appear linked to multiple linkage groups. We chose to keep all scaffolds intact because low-coverage, correctly-placed scaffolds are difficult to distinguish from chimeric scaffolds. The final map from ALLMAPs indicates that the killifish genetic map had an average of 81 cM and 35 MB per linkage group (similar to *D. rerio*) for 24 linkage groups (Woods *et al.* 2000). We estimate an average, genome wide recombination rate at 2.34 cM/MB (Bradley *et al.* 2011).

### Map comparisons

**Figure 3:**
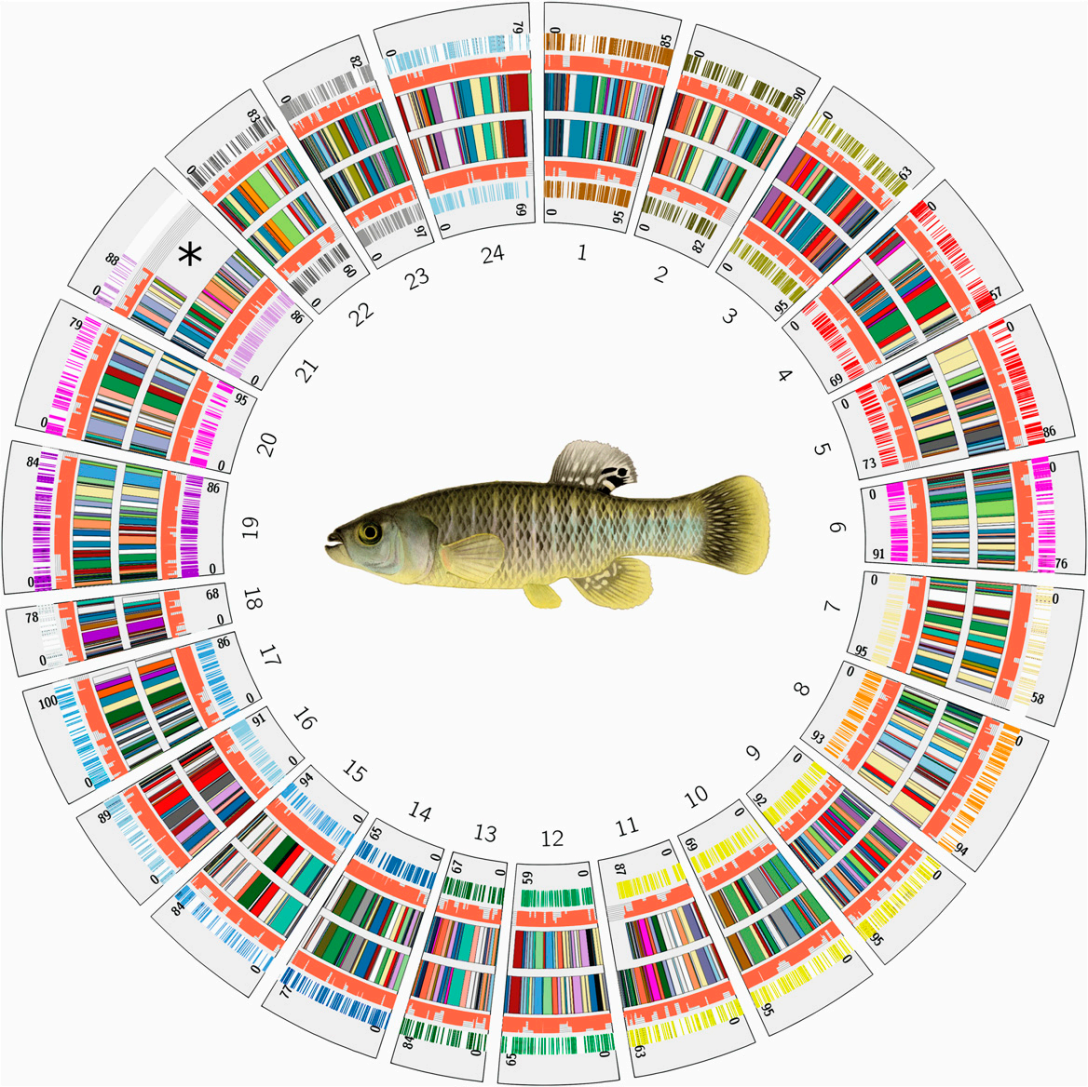
Karyotype and comparison of scaffold ordering and marker density between independent (sex-specific) maps plotted by physical position of scaffolds. Male- (ring of 24 multi-colored LG segments in plot marked by *) and female-specific scaffold orderings (inner ring of 24 multi-colored LG segments). Each linkage group scaffold order is plotted by concatenating the physical size of scaffolds (smaller colored segments in each of the 24 LGs) to emphasize scaffold order rather than the centi-morgan position (cM). The orange bar plots along the scaffolds in each linkage group (left and right of the rings containing scaffold orders) indicate the number of markers (between 1 and 5, fully orange bar is 5 markers used) used for ordering and orienting each scaffold. The outermost track for each linkage group (colored by chromosome) is the position of RAD-Seq markers along each scaffold. Numbers on each group indicate mapping length of each LG in cM. Scaffold orders appear highly congruent (two rows of inner bars colored by scaffold ID) and were independently mapped using pairs of markers that were heterozygous in one sex and homozygous in the other (sex-specific markers). *The differences between the number of scaffolds in the male and female map at LG21 could suggest a heteromorphic chromosome. However, LG21 has a similar total centimorgan length between each map (88 and 86 cM) and share common scaffolds at both ends of the LG, which is typical of homomorphic autosomes (see Figure S3A).

To further examine the quality of the map, we compared the independent male and female genetic maps, and a previously generated microsatellite map (Waits *et al.* 2016). After applying the agglomerative clustering and mapping algorithm, the order of scaffolds appeared highly congruent between sex specific maps (Figure 3). However, we did observe differences in the number and order of some scaffolds in two linkage groups (LGs). For example, the marker density and the number of scaffolds with informative markers in linkage group 21 (LG21) was lower in the male map (see asterisk in Figure 3). This linkage group contains a region of very low recombination, despite a similar total mapping distance between the homologous chromosomes (88 cM in the Male, 86 cM in the Female, Figure S3A). However, the linkage group appears corrected in the consensus map. After merging the maps with ALLMAPs, the consensus map is congruent with the female map that contained a majority of LG21’s markers. When ortholog order is compared with closely related Atherinomorph species (as discussed below), the consensus map for LG21 showed highly conserved synteny with the homologous platyfish chromosome (XM18), with the exception of two transpositions from either chromosomal arm to the other (Figure S3B). The translocation on the lower arm of LG18 is likely real because the region contains multiple scaffolds that are syntenic in both species and medaka. The homologous chromosome LG13 in medaka (Figure S3B, right) does not share this rearrangement, indicating that this inversion has occurred in the platyfish lineage after divergence from killifish.

Sex-specific recombination rates are common in fish and a dramatic reduction in recombination rate accompanied by differences in homologous chromosome structure could indicate a heteromorphic sex determination system (Singer *et al.* 2002). Both LG21 and LG5 differed in scaffold order between the sexes and contained some regions of low combination in the male map (Figures S3A, S1B respectively). Whole-genome re-sequencing of hundreds of individual *F. heteroclitus* (Reid *et al.* 2016) revealed markers that significantly associated with sex on scaffolds that mapped to LG5. Teleost fishes possess a variety of sex determination systems (Schartl 2004; Mank *et al.* 2006; Sandra and Norma 2010) and further study is required to identify candidate mechanisms for sex-determination in *F. heteroclitus*.

**Figure 4:**
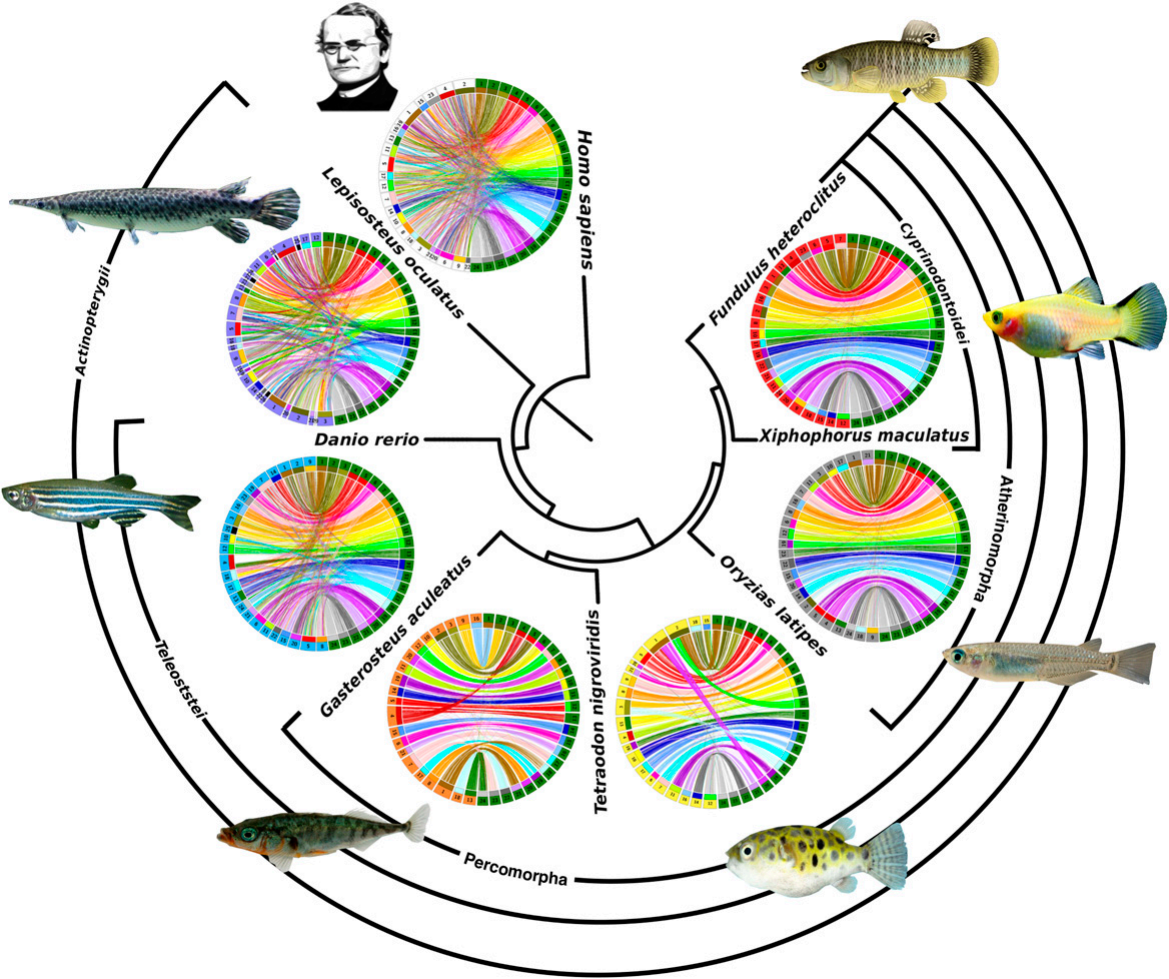
Comparisons of conserved synteny within a phylogenetic framework. Phylogenetic relationship of each species (reference genomes that were mapped to chromosomes) to killifish, indicated at center, increases in distance clockwise from *Fundulus heteroclitus (top right)*. Killifish LGs (green segments at right in each Circos plot) are ordered (1-24) and compared to mapped chromosomes in each species. Colored lines (colored by killifish LG number) in each plot connect the positions of one-to-one ortholog genes between the species. Chromosomes around each Circos plot are ordered by the largest number of orthologs relative to each killifish LG. Highly conserved synteny and orthology (nearly all lines on a chromosome connect to a single killifish LG) was used to identify the homologous chromosomes in percomophs. For larger Circos plots that identify homologs in each species, see supplemental File S6. Fish images were reproduced under the Creative Commons License (File S9).

**Figure 5:**
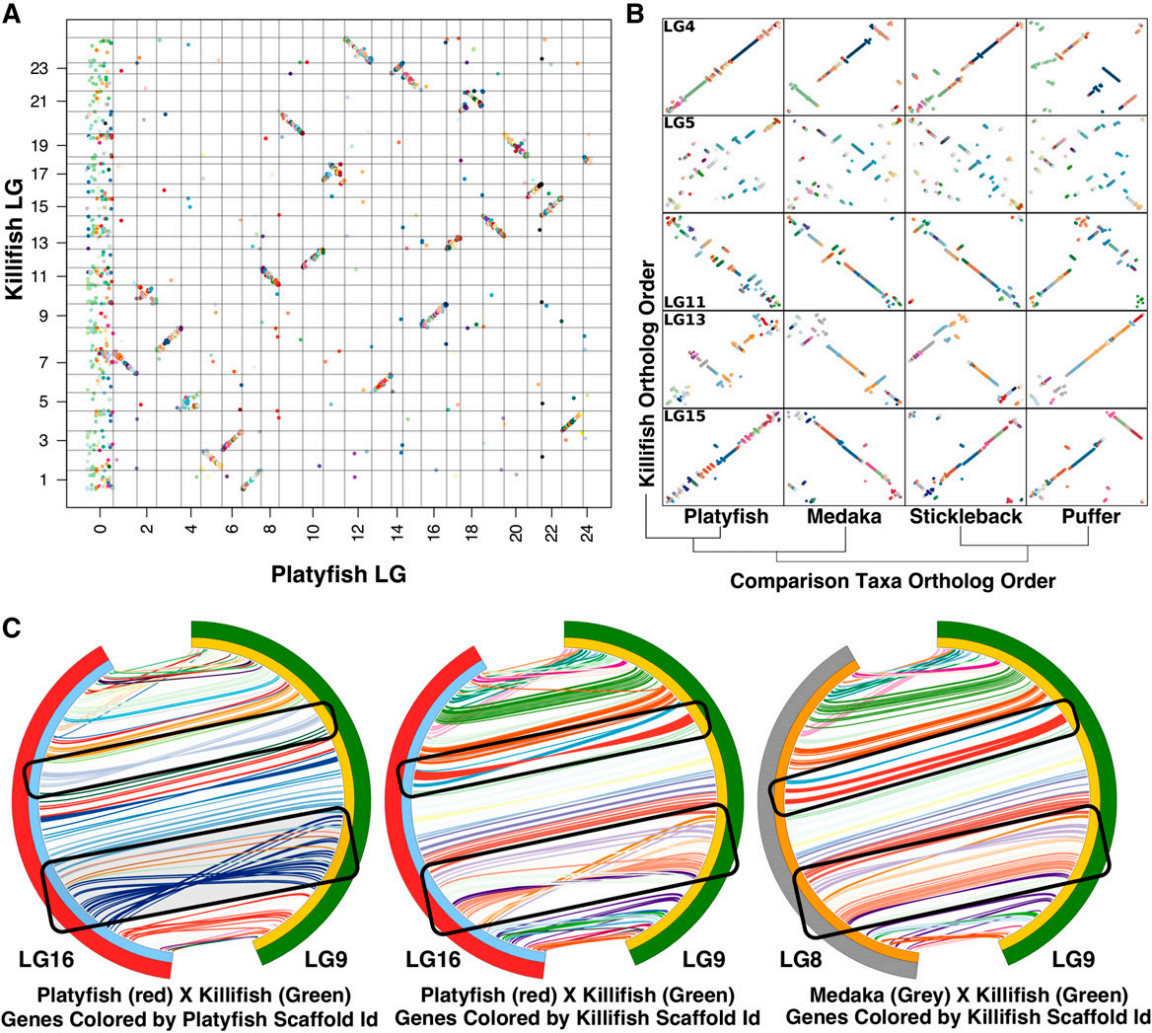
Synteny comparisons with platyfish and medaka. A) Oxford plot for one-to-one ortholog genes (colored dots) between killifish and closely related platyfish. Genes are colored by killifish scaffold identity and ordered by both linkage group and physical position in each reference genome (Killifish along Y-axis, platyfish along X-axis). Homology is conserved at the LG (or chromosome) level if few genes are translocated/mis-assigned (lone points that have mapped to a different chromosome) and if physical positions of orthologs correlate between the homologous chromosomes (tendency for genes to fall along the diagonal in each LG). B) Example killifish LGs and the correlation of physical positions of ortholog in each percomorph. Orthologous genes are colored by killifish scaffold ID (dots, non-homologous LGs left out). Scaffolds that we mapped are in the same relative position in the comparison species if orthologs of the same color (scaffold ID) fall on the diagonal. LGs that contain the same order of scaffolds but contained multiple ‘micro-inversions’ within the same killifish scaffold ID (example in B, LG15 compared to the homologous platyfish chromosome) were considered as possible misoriented scaffolds. C) Killifish chromosome 9 (green) and homologous platyfish chromosome (16, red) and medaka chromosome (8, gray) with orthologous gene physical positions connected by lines that are colored by either platyfish (left Circos plot) or killifish scaffold ID (middle and right Circos plot). Possible mis-orientations were further identified by inversions that appear to be perfectly delimited by scaffold identity in one comparison (ex: large blue platyfish scaffold), but not in the more distantly related comparison (ex the same region of the LG compared to medaka positions) and are not delimited by a single scaffold ID.

Previously, a genetic map was constructed for *F. heteroclitus* using 229 microsatellite and SNP markers which ordered approximately 25% of the genome sequence (Waits *et al.* 2016). Our RAD-seq map extends the proportion of the reference genome included in the map to 83.7%. We compared the consistency of linkage group assignment between the two maps. Most microsat map loci fell on scaffolds with good RAD-Seq coverage in the male and female maps. Twenty-six of the 240 microsat map markers aligned to one of the 216 scaffolds that were missing RAD loci, and these scaffolds tended to be smaller than 0.3 MB, but did include a 1.1, 3.8, and 5.0 MB scaffold. Half of all RAD-seq LGs corresponded exclusively to only one LG from the microsat map. The LGs that contained markers that were differently assigned between the two maps contained a majority of markers that we assigned to the same LG (homolog), but also grouped 1-2 markers that were from different microsat linkage groups, indicating errors in one of the maps. Our data indicate that 19/216 markers from the microsat map may have been incorrectly assigned to linkage groups, and/or these scaffolds contain assembly errors. Scaffolds that were reassigned in the RAD-Seq map are highlighted in File S5. Our data indicate that the small LG18 in the microsat map is actually part of the larger LG6. What we designate as LG18 in the RAD-seq map does not have a homologous LG in the microsat map, indicating that the microsat map failed to sample markers on this chromosome. The 3 markers making up microsat map LG23 were re-assigned to the RAD-seq map LG17 (supported by 26 markers). The microsat map included markers for LG23, but they were mis-assigned to two other linkage groups. Both of these corrections are supported by more markers in the RAD-seq map and highly conserved synteny of the RAD-seq map with other fish maps (Figures 4, 5; File S5). Other than LGs 18 and 23, all LGs have a clear homolog between the RAD-Seq and microsat maps.

### Orthology and synteny analysis

To further examine the quality of the RAD-seq map, we evaluated the shared gene order (synteny) of 1-to-1 orthologs between *F. heteroclitus*, six fish species, and human. We visualized synteny between these genomes within a phylogenetic framework for additional evidence of the order and orientation of scaffolds in the genome assemblies, and to examine patterns of karyotype evolution. Other sequenced Atherinomorphs have 24 linkage groups with clear chromosomal (1-to-1) homology (Fisher and Rachlin 1972; Naruse *et al.* 2000; Walter *et al.* 2004; Amores *et al.* 2014). Our map also grouped killifish scaffolds into 24 linkage groups that are each homologous to an Atherinomorph chromosome, which indicates that our agglomerative clustering approach reliably assigned scaffolds to chromosomes. Similar to the platyfish-to-medaka map comparison (Amores *et al.* 2014), we detected very few potential inter-chromosomal rearrangements per linkage group between species in this clade, with the number of putative translocations ranging from zero (LG12 orthologs appear exclusively on platyfish 10 or as unmapped in platyfish, with only one apparent transposition in medaka) to 10 with an average of 3.97 per chromosome (Figure S4). Among Atherinomorphs, an average of 2.2% (143 of nearly 7,000 genes) of all putative one-to-one orthologs are designated as orthologous to a gene on a chromosome other than the homologous chromosome. The translocations that do appear tend to include a single gene model (singletons) rather than major structural rearrangements that carry multiple syntenic genes. Singletons are either genome assembly errors, errors in assigning orthology (older paralogs and true ortholog loss), or small real translocations in each branch. At least 10/265 singleton genes are found to be translocated in all teleosts relative to killifish, which indicates killifish specific error in assigning orthology or translocations. Interestingly, singletons appear at an even lower rate in the more distantly related Percomorpharians (∼3.3 singletons per chromosome) but are elevated in zebrafish (at least 5.16/chromosome), gar and human (Figure S5-6). Transposable element activity and repetitive sequence is related to single gene transpositions (Montgomery *et al.* 1991). Zebrafish transposable element diversity and abundance is the highest among the teleosts in our analysis (Sotero-Caio *et al.* 2017). This may indicate that singletons in more distantly related taxa could be attributed to a combination of errors and real translocations.

We also observed previously described chromosomal rearrangements that are responsible for the differences in karyotype number among some teleost clades (Jaillon *et al.* 2004; Roesti *et al.* 2013). The karyotype number and genome structure of ancestral Teleostei is likely 23-24 chromosomes and is highly conserved in medaka (Kasahara *et al.* 2007). The reduction of chromosome number in Percomorpharians (stickleback and pufferfish, n = 21) occurred after the divergence from the Atherinomorph lineage (Kai *et al.*, 2011). Our map recovered the independent chromosomal fusions in each lineage and the shared synteny with their ancestral homologous chromosome segments (Figure S7A). Relative to Percomopha, the zebrafish lineage (n = 25) diverged earlier in the Teleost radiation. Relative to killifish and the ancestral 24 teleost chromosomes, the zebrafish genome experienced an increase in independent inter- and intra-chromosomal rearrangements (Kasahara *et al.* 2007, Figure S5 A-E). However, as with previous comparisons (Amores *et al.* 2014), the accumulation of these complex rearrangements and an increase in singleton translocations makes it difficult to infer the events that shaped the zebrafish karyotype compared to *F. heteroclitus*.

At the broader phylogenetic scale, we observed expected patterns of highly conserved chromosome homology and synteny between closely related species that deteriorates with phylogenetic distance (Figure 4). Much structural genome variation has evolved over the more than 300 million years since sharing common ancestry with spotted gar. However, we recovered the characteristics of the whole genome duplication and reciprocal gene loss that occurred in the teleost linage after the divergence from the lineage leading to spotted gar (Jaillon *et al.* 2004; Amores *et al.* 2014). For example, dot-plots comparing killifish to human and killifish to spotted gar chromosomes shows that almost all 1-to-1 orthologs for a given region in these taxa are distributed primarily on two *F. heteroclitus* chromosomes (*e.g.*, gar LG8 and human LG14, Figure S6). Circos plots of the gar and duplicated killifish chromosomes (ohnologs) show that the 2:1 homology often spans entire chromosomes and does not indicate fission (Figure S7B, chromosome split with syntenic segments of homology to different chromosomes). After duplication, ohnologs tend to evolve such that one of the two duplicate genes are lost (reciprocal gene loss) leading to the overlapping pattern of orthologs that we observe (Figure S7B; (Woods *et al.* 2005).

Genome-wide visualizations indicate that orthologous genes appeared ordered the same between platyfish and killifish (Figure 5A). However, closer inspection at the chromosome level revealed very short regional inversions (termed micro-inversions here, Figure 5B). In fact, some chromosomes appear to be dominated by micro-inversions (*e.g.*, LG15, Figure 5B), while others are almost completely syntenic with killifish (no micro-inversions, *e.g.*, LG4, Figure 5B). It is possible that these micro-inversions are mistakes in scaffold orientation within the linear ordering of a linkage group. If micro-inversions are bounded by the ends of a scaffold, then this is suspicious and unlikely to be a real inversion, and more likely to be a mistake in scaffold orientation within the map. To examine this, we generated synteny plots where ortholog connections are colored by scaffold identity (Figure 5C). We discovered that inversions were often delimited by scaffold boundaries in either the platyfish or killifish map, suggesting that they are likely mis-oriented scaffolds. To further test whether micro-inversions are mis-orientations, we compared gene order in a third species. We predicted, for example, that mis-orientations in platyfish would appear as 1) a short region negatively ordered to killifish, but not mis-ordered between killifish and medaka, and 2) delimited by platyfish scaffold ID. Scaffolds that meet this criterion are found in both platyfish and killifish. The scaffolds highlighted in the black box in Figure 5C are examples of likely scaffold mis-orientations in platyfish. In some cases, synteny appears much more strongly conserved between more distantly related taxa and killifish (*e.g.*, LGs 11, 13, 15, Figure 5B) than between killifish and platyfish (LG14, Figure 5B). This indicates either an increase in rearrangements in the platyfish lineage or an elevated rate of scaffold mis-orientation in these regions. We consider the latter explanation more likely. Our comparison suggests mapping data tend to order scaffolds correctly within a chromosome, but that scaffold mis-orientations within that arrangement are common. These observations suggest that caution should be observed when interpreting fine scale structural variation between genomes assembled with recombination mapping alone. Mis-orientations would bias analyses toward higher levels of intra-chromosomal inversions and rearrangements. For chromosomes that have a higher rate of rearrangements such as the putative sex chromosome LG5 (Figure 5B), it would be impossible to distinguish micro-inversions from intra-chromosomal rearrangements. Longer scaffolds, for example generated from long-read sequencing, are likely less susceptible to mis-orientation with recombination mapping.

A quality mapped reference genome enables comparison of genome structure between species. A quality map is also an important resource in evolutionary and ecological genomics studies, such as those that aim to establish genotype-phenotype relationships and the genetic basis of local adaptation via signatures of selection. Genotype-phenotype association mapping (*e.g.*, GWAS, QTL mapping, admixture mapping) reveals the identity and number of genomic regions that associate with phenotypic variation among individuals. Adaptation genomics seeks to identify regions of the genome showing signatures of natural selection within and between populations, where regions can be large because of linked selection (Hill and Robertson 1966; Sawyer *et al.* 2007). A good reference genome, where scaffolds are correctly ordered and oriented, helps to distinguish the boundaries of genomic regions that are diverging by natural selection. In particular, signatures of very recent or strong selection may be manifest across wide regions of a chromosome that may span multiple scaffolds. For example, some signatures of strong and recent selection during adaptation to extreme pollution in killifish were greater than 1 Mb (Reid *et al.* 2016). In a highly fragmented or mis-ordered assembly, this footprint of selection could be expected to span many scaffolds, inflating the estimated number of genomic regions that were targets of selection, and potentially altering conclusions about the polygenic nature of adaptive phenotypes.

## Conclusions

Our map improves the reference genome of killifish *Fundulus heteroclitus* to further its utility for studies in ecological and evolutionary genetics. We resolved a large portion (∼84%) of the reference genome into 24 linkage groups (chromosomes). We describe the rationale for each of our empirical data filtering steps to provide explicit guidance for the selection of study-specific empirical quality filtering thresholds that may be useful for others. This careful filtering of read and genotype data dramatically improved our resulting maps. We evaluated the quality of our map by comparing the synteny of 1-to-1 orthologous genes to other mapped genomes within a phylogenetic framework. We recovered signals of whole genome duplications, fusion/fission and rearrangements that have been described in other comparative analyses, confirming the accuracy of our map. We also found that scaffold misorientations within linkage groups are common and suggest caution when interpreting fine scale variation between closely related species due to these linkage mapping artifacts. This well-assembled genetic map will enable the next generation of quantitative genetic and population genetic studies in *F. heteroclitus*.
